# Perspectives in poverty and mental health

**DOI:** 10.3389/fpubh.2022.975482

**Published:** 2022-08-04

**Authors:** Derin Marbin, Stefan Gutwinski, Stefanie Schreiter, Andreas Heinz

**Affiliations:** ^1^Department of Psychiatry and Neurosciences, Charité – Universitätsmedizin Berlin, Corporate Member of Freie Universität Berlin, Humboldt-Universität zu Berlin, and Berlin Institute of Health, Berlin, Germany; ^2^Department of Psychiatry of University Hospital Charité in St. Hedwig Hospital Berlin, Germany

**Keywords:** income inequalities, poverty & inequality, mental health, homelessness and mental health, mental illness

## Abstract

In recent years, different forms of poverty and their interaction with mental illness have been in the focus of research, although the implementation of action in mental health care and policy making so far is scarce. This perspective article offers different perspectives of poverty and its reciprocal association with mental illness and outlines possible future research and policy implications. We will approach the topic of poverty from various levels: On a micro-level, focusing on absolute poverty with precarious housing and malnutrition. On a meso-level, on neighborhood-related poverty as a factor in individuals' mental illness. On a macro-level, on effects of income inequality on mental health. In several studies, it has been shown that on each level, poverty has a profound impact on mental health, though it must be noted that in some fields, research is still scarce. In the future, an inter- and transdisciplinary approach is of considerable importance, since poverty and its impact on mental health should be addressed from different perspectives, reaching from targeted programs for individual groups (e.g., homeless people) up to national policy measures.

## Introduction

The Global Burden of Disease study estimated that, in 2017, 792 million people worldwide reported impaired mental health, which represents almost 11% of the global population (1). According to The World Health Organization, mental health conditions produce economical losses of one trillion USD, with depression being the leading cause of ill health and disability (2, 3).

Especially people living in poverty are unequally affected by mental illness (4). The United Nations (UN) defines poverty as “…a condition characterized by severe deprivation of basic human needs, including food, safe drinking water, sanitation facilities, health, shelter, education and information. It depends not only on income but also on access to social services” (5). According to the World Bank, the extreme poverty line concerning daily expenses is under 2.15$ based on 15 national poverty lines from some of the poorest countries in the World (6). National poverty lines vary depending on the respective costs to cover one's basic needs, e.g., in the European Union, someone earning <60% of the median income is “at-risk-of-poverty” (7). Relative poverty on the other hand, is defined as not having enough material, cultural, and social resources and thus be excluded from a lifestyle, which other individuals from the respective country can maintain (8).

Poverty has increased globally since the COVID-19 pandemic. The United Nations University reported that after 30 years of decline of poverty, the global pandemic could lead to an increase of global poverty by 8% since 2020, with rates being three times higher in rural compared to urban areas (9).

In this perspective article, we focus on the impact of poverty on mental health in high-income countries. We discuss effects of poverty on a micro-, meso- and macro-level and outline implications for future research and mental health policies. On the micro-level, we discuss individual characteristics including material, psychosocial, and behavioral risk factors, using the example of precarious housing and malnutrition. On a meso-level, we refer to community- and neighborhood-related circumstances with a focus on local poverty, social exclusion and discrimination, and their respective effects on individual mental health. On a macro-level, we focus on the association between income inequality within a nation and the national mental health burden.

## Absolute poverty in high-income countries

Rates of poverty in the Organization for Economic Co-operation and Development (OECD) countries vary between 4.9% in Iceland as the lowest rate up to 19.9% in Costa Rica, whereby the highest poverty rate among high-income countries can be found in the United States with 17.8% (10). One subgroup affected by poverty in high-income countries are people exposed to homelessness. With an estimation of 1.9 million persons without a home, and increasing numbers in countries like the United States, United Kingdom and Germany (11), several research and policy initiatives focus on the interrelation between mental health and living conditions in poverty.

In a systematic review and meta-analysis of 39 studies with altogether 8,049 homeless persons in Germany, a pooled prevalence of a current mental illness of 76.2% (95% CI 64.0–86.6) was reported, with alcohol dependence being the most common disorder (pooled prevalence 36.7% [95%CI 27.7–46.2]) (12). In a systematic review and meta-regression among western countries, 29 studies with 5,684 individuals reported a pooled prevalence of 37.9% (95%CI 27.8–48.0) for alcohol dependence and 24.4% (95%CI 13.2–35.6) for drug dependence (13). Substance use among homeless people is often heavily stigmatized, and even healthcare professionals have displayed a rather negative attitude toward patients with substance use, which might have an influence of the self-esteem and empowerment of patients and thus affect treatment outcomes such as treatment completion (14). Beyond drug use, adverse life events, suicidality and mental illness are important predictors of becoming homeless (15). There is a complex interplay between homelessness and mental illness, with mental health challenges increasing the risk of homelessness, and homelessness promoting poor mental health including depression and suicidality (16).

Persons with debts and substantial loans represent another group affected by poverty in high-income countries. In Western countries like Germany, 8.6% of the general population have debts that cannot be cleared because of insufficient income and assets (17). Again, persons with mental health issues are disproportionately affected by debt. In a study with 486 psychiatric patients, 55.1% had outstanding debts, loans, or unpaid bills, of which more than a third (36.3%) reported debts between 10.000 and 99.999*e* (18). Here, binary regression analysis identified younger age and substance use disorders as being significantly associated with outstanding debts [OR 0.98 (95%CI 0.96–1.00) and OR 2.41 (95%CI 1.48–3.92)] (18).

Another important aspect of absolute poverty is insufficient nutrition. There is an increasing focus on the interaction between food security and mental health as main sources of global mortality and disease (19). For example, Fang et al. conducted a study during the COVID-19 pandemic among 2,714 low-income participants in the United States and observed that food insecurity was associated with a 257% higher risk of anxiety [as measured by the GAD-7; OR 3.57 (95%CI 3.01–4.23)] and a 253% higher risk of depression [measured by PHQ-9; OR 3.53 (95%CI 2.99–4.17)] (20). Insufficient nutrition is a risk factor, while income stability was detected as a protective factor for depression [OR 0.77 after adjusting for income stability (95%CI 0.66–0.91)]. Especially respondents with children were identified as the most vulnerable subgroup. This evidence is supported by findings of the Global Burden of Disease Study 2019 (21), which reported that child and maternal malnutrition was one of the leading risk factors for disability-adjusted life-years. This study emphasizes the pivotal importance of targeted nutritional programs as a part of women's health in the context of mental health care. It also implicates that reaching out to vulnerable groups should not be restricted to mental health care settings, but that including interventions in the general community is essential, where e.g. mothers can be provided with adequate resources.

## Poverty in the neighborhood

People living in socially underprivileged and poor city areas suffer more often from mental health conditions like depression, anxiety and psychosis than persons living in high-income neighborhoods (22–24). For example, Fone et al. reported that regional income inequality was significantly associated with more common mental disorders [measured by the Gini coefficient^1^ and the Mental Health Inventory MHI-5; Odds Ratio = 1.13 (95%CI 1.04–1.22)] (22). In addition, more than half of the world's population live in cities, and the continuous urbanization of city areas lead to an aggravation of community-level risk factors for mental health conditions, including physical environmental challenges or chronic stress exposition, even though city residents have more access to education and healthcare (25, 26). Accordingly, in a meta-analysis, Vassos et al. reported a 2.37 higher risk for schizophrenia for people living in urban environments compared to rural environments (24).

During the last decade, the effect of neighborhood-related factors (including social cohesion, income deprivation as well as traffic or air pollution) on the mental health status of residents has gained attention (27). It has been suggested to interpret data on individual risk factors including income and education with respect to their interaction with the social environment (28, 29). The relationship between factors on a neighborhood level and individual outcomes can be complex (30), with multiple, potentially bidirectional pathways to explain the association between community-level and individual factors. In spite of these potential complexities, there is evidence that poverty in the neighborhood is associated with poor mental health (measured by the General Health Questionnaire GHQ-28) above and beyond the effects of individual education or income (31). This effect was even more pronounced among persons with a minority status (persons with a Turkish migration background in Berlin, Germany), and again independent of individual factors like age or income (beta = 1.12, Standard Error = 0.26, *p* < 0.001) (31). This observation is supported by a longitudinal cohort study with 1,120 participants in New York, which observed that the socioeconomic status (SES) of the neighborhood was associated with the incidence of depression, independently of the individual SES (28).

Next to economic deprivation, social-interactive aspects of the neighborhood should be taken into account. For example, a longitudinal multilevel analysis with 4,426 participants over the course of 7 years examined quality of life and mental health (36-Item Short Form Survey and Mental Health Inventory-5), neighborhood deprivation (gross household income) and social cohesion, which refers to a sense of belonging and solidarity within a community (Buckner's Neighborhood Cohesion Scale) (32). This study reported a negative association between neighborhood challenges due to poverty on the one hand and low levels of mental health and quality of life on the other, again after adjusting for individual socio-economic risk factors and transitions in life events. Interestingly, a protective effect of solidarity and social cohesion on this association was found. These findings suggest that individual mental health is substantially influenced by local poverty, and that solidarity and social support is of key importance for mental wellbeing (33–35).

Data on mediating processes between neighborhood poverty and mental wellbeing are rare and may include aspects of family and peer support structures (4, 30). Lack of family and neighborhood resources can lead to more stress, social isolation, discrimination and susceptibility for mental disorders (31). Mental illness, on the other hand, can increase stigmatization, social exclusion and marginalisation (4, 36). Also, income in people with mental health conditions is usually decreased, reducing resources and leading to a higher probability to live in a poor neighbourhood (4). Physical aspects of neighborhoods may also play an important role for mental wellbeing. In Greenwich, UK, it has been shown that physical environment and mental wellbeing are associated with each other on many domains (37). In this study, living conditions for persons registering in the lowest quartile for mental health were characterized by neighborhood noise [OR 2.71 (95%CI 1.48–4.98)], feeling overcrowded in the home [OR 1.42 (95%CI 1.42–3.48)], being dissatisfied with access to green open spaces [OR 1.69 (95%CI 1.05–2.74)], and feeling unsafe to go out in the day [OR 1.64 (1.02–2.64)].

Prospective studies are required to disentangle the interaction of individual, environmental and community level effects on mental health.

## Income inequality and its effects on mental health

Looking at relative poverty on a macro-level, general effects of income inequalities on mental health have to be considered, as numerous studies have shown the effect of income inequality on overall health and mortality in high income countries (38–41). In their list of 17 sustainable development goals, the UN declared reducing income inequalities within and among countries as one aim (5). In the last decades, income inequality has dramatically increased in Western industrialized countries (42). According to the epidemiologists Pickett and Wilkinson, income inequality is linearly associated with higher rates of mental illness in high income countries (38, 43). Industrialized countries with high income inequality, like the United States (measured by the ratio of income among the wealthiest compared with the poorest 20% in each country) showed a high index of health and social problems (e.g., life expectancy, mental illness, homicides, distrust, social mobility) (44). In 2022, Tibber et al. included 42 studies with data from 7,744,469 participants and found higher income inequality to be associated with poor general mental health, depression and psychosis in adults (45). Likewise, a review of 26 studies from mostly high-income countries reported a positive relationship between income inequality and depression, with greater impact for women and low-income subpopulations (41). Regarding schizophrenia, Burns et al. investigated incidence rates across 26 mostly high-income countries and found a positive relationship between income inequality, measured by the Gini coefficient, and the incidence rate of schizophrenia [beta = 1.02; *Z* = 2.28; *p* = 0.02; (95%CI 1.00–1.03)]: for every point in income inequality increase, a two-point increase in incidence rate of schizophrenia followed (46). After correction for potential confounders like rates of urbanization, Gross Domestic Product per capita, migrant population and unemployment rate, the effect still remained significant (46).

Regarding mortality caused by mental health conditions, suicide rates among young men in England and Wales increased over the period of 1950–1998, which was associated with an increase in income inequality and divorce and a decline in marriage (47). A longitudinal study from Canada investigated mental health in 2,461 mothers during pregnancy and after birth and found a significant interaction between income inequality measured by the Gini coefficient and anxiety symptoms, but not depressive symptoms (48). In a register-based cohort study with 1,354,393 children, mental disorders were three–four times more prevalent in children who had parents in the lowest income percentiles (49). Differences were detected concerning attention-deficit hyperactivity disorder in boys and depression and anxiety in girls. Wilkinson and Pickett elaborated on the importance of early childhood interventions to reduce developmental risk factors for health (50).

To explain these findings, several causal hypotheses are discussed. For example, the Social Capital Hypothesis suggests high income inequality affects (mental) health because of a breakdown of social capital, which includes social trust and safety, a sense of belonging, and participation (38, 39, 51). The Status Anxiety Hypothesis states that high income inequality fuels a feeling of inferiority because of high status competition and comparison, which causes chronic stress (52). In his book, “Status Syndrome,” Marmot states that position in hierarchy, which is linked to control over life and social engagement, is the most important factor for health inequalities, rather than factors like genetics or behaviour (53, 54). This hypothesis is supported by a study using data of 34,000 study participants across 31 European countries, conducting multi-level models with the Gini coefficient, several sociodemographic factors, and status anxiety, as assessed by the multi-scale question “some people look down on me because of my job situation or income” (52). In this latter study, status anxiety was inversely associated with income rank; moreover, status anxiety was also lower in countries with lower income inequality. Beyond status anxiety, perception of unfair income distribution may be particularly pronounced in cities with relatively segregated high and low income neighborhoods and exposure to dramatic differences in resources and privileges (55). Again, longitudinal studies can help to disentangle complex interactions between individual risk and resilience factors, community resources and environmental challenges.

## Discussion: Future policy implications

The reviewed studies suggest that a person's mental health is not only and even not primarily explained by individual risk factors, but includes closely related community and environmental processes that reflect social differences and justice. In this context, Amartya Sen's capability framework emphasizes the responsibility of the society to contribute to each of its members' self-fulfilment (56). Together with the conceptual work of Michael Marmot (54), these considerations shift the focus away from stigmatizing socially and economically excluded individuals toward a reflection of multidimensional processes within a society, which steer vulnerable people in the direction of poverty and compromised somatic as well as mental health.

Among many conceptual frameworks, the World Health Organization formulated three levels of policy approaches in their call for action on the social determinants of health, to tackle general health inequities (57). Based on this, we elaborate on specific mental health and poverty strategies and recommend:

Targeted programs for groups with a low socioeconomic status, including homeless persons with mental illness. Financial inclusion of people with mental illness, especially those in unstable housing, should be in the focus of targeted programs. Indeed, our own research showed that in Berlin Germany, about 10.1% of all patients have no bank account, thus severely restricting access to social aid and limiting participation (58). Strategies of permanent supportive housing like Housing First have proven to be effective on housing stability as well as health outcomes (15). Other examples for targeted programs might be nutritional programs for women or young mothers, since child and maternal malnutrition are one of the leading risk factors for disability-adjusted life-years (21).

Policies for closing the gap of social inequalities. Here, scientists from different disciplines should cooperate to assess the impact of poverty on the general mental health of a community, and to disentangle complex interactions on the level of communities, environments and individuals. Our own research emphasizes the impact of local poverty above and beyond individual income (31). This observation supports the implementation and examination of effects of anti-poverty programs including Universal Basic Income (UBI) (59). A study on the effects of basic income provided by cash payments in Finland reported significant improvements in mental wellbeing; mediating factors were associated with a reduction in perceived stigma, more time with family and friends, and a new sense of hope for the future (60). Interestingly, improvements for children were amplified when the payments were given early during their individual development (60). Nevertheless, it should be considered that UBI might also be discriminating toward people with different needs and thus higher living expenses, for example for people with chronic diseases and higher health expenditures. Also, UBI might only be offered to people with a certain citizenship, excluding already marginalized people without any citizenship for example. In light of the effects of income inequality on mental health, economic growth *per se* will not lead to an increased mental wellbeing, and a balanced distribution of wealth should be considered in policy including metal health strategies (61).

Promoting interdisciplinary and participatory research on social interactions in societies. To date, participatory research and peer involvement is widely underrepresented and should play a much more influential role in scientific studies and policies for mental health (62). On a neighborhood level, solidarity and mutual support appears to represent important mediators between neighborhood poverty and individual mental health. It should be an inter- as well as transdisciplinary effort including social sciences to disentangle these processes and their complex interactions. We and others have suggested to promote topics relevant for mental health in all aspects of city planning, including exposure to air pollution, traffic noise, also at nights, provision of green spaces, accessible community centers and spaces for social interaction (25, 26).

To disentangle the complex impact of absolute and relative poverty on mental health, studies should be designed longitudinally and measurements should be included that address mental as well as somatic health, risk factors such as racism and discrimination and potential resilience factors such as solidarity and mutual support. Facing the increasing digitization of health care, the use of digital tools and digital interventions to collect data can be of help with this endeavour (63, 64).

Altogether, an inter- and transdisciplinary approach can promote understanding of the complex and multileveled interactions between individual- and community-based risk factors. The aim is to address mental health in populations with evidence-based public health policies that target social and physical environments and foster solidarity and mutual support. Medical prevention and intervention strategies targeted at the provision of adequate mental health care for persons with mental illness should be complemented by policies that promote social participation and empowerment within societies.

## Data availability statement

The original contributions presented in the study are included in the article/supplementary material, further inquiries can be directed to the corresponding author.

## Author contributions

DM and AH were responsible for drafting and revising the manuscript. DM wrote the first manuscript draft. AH, SS, and SG revised the manuscript. All authors contributed to and approved the final manuscript.

## Conflict of interest

The authors declare that the research was conducted in the absence of any commercial or financial relationships that could be construed as a potential conflict of interest. The handling editor [WF] declared a shared affiliation with the author(s) at the time of review.

## Publisher's note

All claims expressed in this article are solely those of the authors and do not necessarily represent those of their affiliated organizations, or those of the publisher, the editors and the reviewers. Any product that may be evaluated in this article, or claim that may be made by its manufacturer, is not guaranteed or endorsed by the publisher.

## Footnotes

^1^To objectify income inequality, several measures have been established, of which the Gini index is one of the most commonly used (65). It ranges from 0 to 1, with higher index indicating greater income inequality. The calculation relates to the Lorenz curve, which is a graphical representation of distribution of income.
